# Exploration of the mechanisms of HLWDD on skeletal muscle lesions under the influence of diabetes based on bioinformatics analysis and experimental validation

**DOI:** 10.3389/fnut.2025.1586761

**Published:** 2025-12-11

**Authors:** Peng Chen, Huasen Yang, Zhoujing Shi, Likun Du, Yue Yin, Bei Jiang, Boyan Ma

**Affiliations:** 1 First Affiliated Hospital of Heilongjiang University of Chinese Medicine, Harbin, China; 2 School of Basic Medicine Heilongjiang University of Chinese Medicine, Harbin, China

**Keywords:** bioinformatics, type 2 diabetes mellitus, skeletal muscle, HLWDD, apoptosis

## Abstract

**Objective:**

To investigate the molecular mechanisms of Huanglian Wendan Decoction in treating type 2 diabetes mellitus-associated skeletal muscle lesions via integrated bioinformatics and experimental validation.

**Methods:**

T2DM-related skeletal muscle microarray datasets from the GEO database were analyzed to identify differentially expressed genes (*n* = 3309). Apoptosis-related targets (*n* = 887) and T2DM-related targets (*n* = 3106) were retrieved from GeneCards, and intersection analysis yielded 96 shared targets. Protein-protein interaction networks were constructed using STRING and Cytoscape; via CytoHubba, 10 core targets were first screened based on degree centrality. ROC curves were further used to validate the diagnostic efficacy of these 10 core genes, ultimately confirming 5 key targets (EGFR, PTEN, MDM2, TRAF6, and CCL5) with valid diagnostic value. Functional enrichment analysis revealed pathways including cysteine-type endopeptidase activity regulation and pyroptosis, while immune infiltration analysis linked the key targets to immune cell modulation. Molecular docking was used to assess the interactions between HLWDD compounds and the 5 key targets. A T2DM rat model was established using a high-fat diet combined with streptozotocin; biochemical parameters and skeletal muscle morphology were evaluated, and the protein expression of the 5 key targets was analyzed using immunohistochemistry and western blotting.

**Results:**

HLWDD significantly reduced HbA1c, blood lipid, glucose, and renal dysfunction markers (*P* < 0.05), improved skeletal muscle histology, and downregulated core target proteins. Bioinformatics has highlighted the association of core genes with apoptosis and immune responses. ROC analysis demonstrated strong diagnostic potential (AUC > 0.5).

**Conclusion:**

HLWDD alleviated T2DM skeletal muscle injury by modulating apoptosis-related pathways and immune interactions, as supported by multi-omics and experimental validation. This study provides novel therapeutic targets and mechanistic insights into HLWDD for T2DM management.

## 1 Introduction

Diabetes is a prevalent metabolic disorder characterized by persistently elevated blood glucose levels (1). This condition arises primarily from insufficient insulin secretion or reduced insulin sensitivity, leading to impaired regulation of sugar, protein, and lipid metabolism (2). Diabetes can be classified into two main types: type 1 and type 2. Among these, Type 2 Diabetes Mellitus (T2DM) is the predominant form, accounting for more than 90% of all cases (3). The interaction between genetic and environmental factors is crucial for the pathogenesis of T2DM (4). Notably, obesity and aging contribute to a cascade of physiological changes, including chronic inflammation, elevated oxidative stress, abnormal apoptosis, autophagy dysfunction, and imbalance of the gut microbiota (5, 6). Collectively, these changes lead to insulin resistance and a gradual decline in pancreatic β-cell function (7). As the disease progresses, patients with T2DM often develop multisystem complications, such as diabetic nephropathy, retinopathy, and cardiovascular diseases (8).

Skeletal muscle lesions, a key complication closely linked to metabolic dysfunction, also merits attention. Skeletal muscle, one of the largest tissues in the human body, not only supports movement, but also plays a core role in regulating systemic glucose homeostasis (9). It is the major target organ of insulin action, responsible for metabolizing ~80% of circulating glucose (10). In healthy states, skeletal muscle efficiently responds to insulin to promote glucose uptake and utilization; however, in T2DM, this regulatory mechanism is disrupted—chronic low-grade inflammation reduces skeletal muscle insulin sensitivity (11), impairing glucose transport and phosphorylation (12), which exacerbates hyperglycemia. T2DM also causes skeletal muscle structural and functional decline (e.g., reduced muscle mass and strength), further disturbing systemic glucose balance and lowering patients' quality of life (13, 14). Impaired skeletal muscle glucose homeostasis increases the liver metabolic burden, potentially reducing hepatic glycogen synthesis and promoting gluconeogenesis, thus forming a vicious cycle of T2DM progression. Therefore, protecting skeletal muscle health is critical for T2DM prevention and treatment. Notably, the disease is trending toward earlier onset, prolonged course, and diversification of complications, severely impacting patients' quality of life and socioeconomic conditions (15). Therefore, the prevention and control of T2DM has become a significant global public health challenge urgently requiring solutions (16).

In traditional Chinese medicine (TCM) theory, although there is no direct equivalent term for diabetes as in modern medicine, based on clinical manifestations such as excessive drinking, excessive eating, and weight loss, it can be categorized under “Xiaoke” (wasting-thirst) syndrome. According to TCM, the development of diabetes is closely related to excessive Yang energy, internal heat accumulation, and depletion of Yin fluids, which collectively lead to the disease symptoms Huanglian Wendan Decoction (HLWDD), first recorded by physician Lu Tingzheng in the Qing Dynasty classic Liu Yin Tiao Bian, consists of eight herbs: Coptidis rhizoma, Caulis bambusae in taenia, Aurantii fructus, Pinellia ternata, Citrus reticulata, Glycyrrhiza uralensis, Zingiber officinale, and Poria cocos. This prescription functions to clear heat and dampness, regulate Qi, and resolve phlegm, and has traditionally been applied to treat “damp-heat” syndromes. Recent pharmacological studies have suggested that HLWDD may exert beneficial effects in metabolic diseases, such as T2DM, through multi-target regulation. Modern pharmacology has revealed that the main bioactive constituents of HLWDD, notably alkaloids, flavonoids, and organic acids, exhibit distinct antidiabetic properties. For instance, alkaloids in Coptis chinensis (e.g., berberine) significantly reduce fasting blood glucose and glycated hemoglobin levels in diabetic mice (17). while flavonoids in Citrus reticulata enhance insulin sensitivity and modulate gut microbiota composition (18).

Moreover, the five major compounds—neohesperidin, hesperidin, naringin, palmatine, and ferulic acid (19)——collectively contribute to HLWDD's therapeutic profile. Neohesperidin from citrus peels lowers blood glucose and serum lipid levels while alleviating insulin resistance (20); hesperidin, a citrus flavonoid, inhibits macrophage infiltration and improves high-fat diet-induced hyperglycemia (21, 22); naringin (from pomelo/grapefruit) reduces blood lipids and inhibits digestive enzymes (e.g., α-glucosidase) to limit glucose absorption (23–26); palmatine (from Corydalis and Coptis) enhances insulin sensitivity and regulates glucose-lipid metabolism (27–29); and ferulic acid (from vegetables and grains) exerts antioxidant effects and upregulates insulin signaling molecules (30–33). Although the herbal components and key chemicals of HLWDD have been proven effective against T2DM, their specific mechanisms of action (especially in alleviating skeletal muscle lesions) need further elucidation. Recent studies suggest that HLWDD may exert its effects through several mechanisms: improving pancreatic β-cell function to enhance insulin secretion (34); increasing glucose utilization to lower blood sugar (35); inhibiting inflammation to reduce insulin resistance (36); modulating gut microbiota composition (37), and improving glucose-lipid metabolism, particularly in T2DM patients with damp-heat syndrome (38). Collectively, these findings indicate that HLWDD exerts multi-component, multi-pathway, and multi-target regulatory effects, making it a promising TCM formula for T2DM management. However, despite preliminary insights, the precise molecular targets and pathways, especially those associated with skeletal muscle lesions, require further elucidation. This study therefore aims to explore the pharmacological mechanisms of HLWDD using integrated bioinformatics and experimental validation, thereby providing new evidence for the modernization and scientific understanding of traditional Chinese medicine.

**Figure 1:**
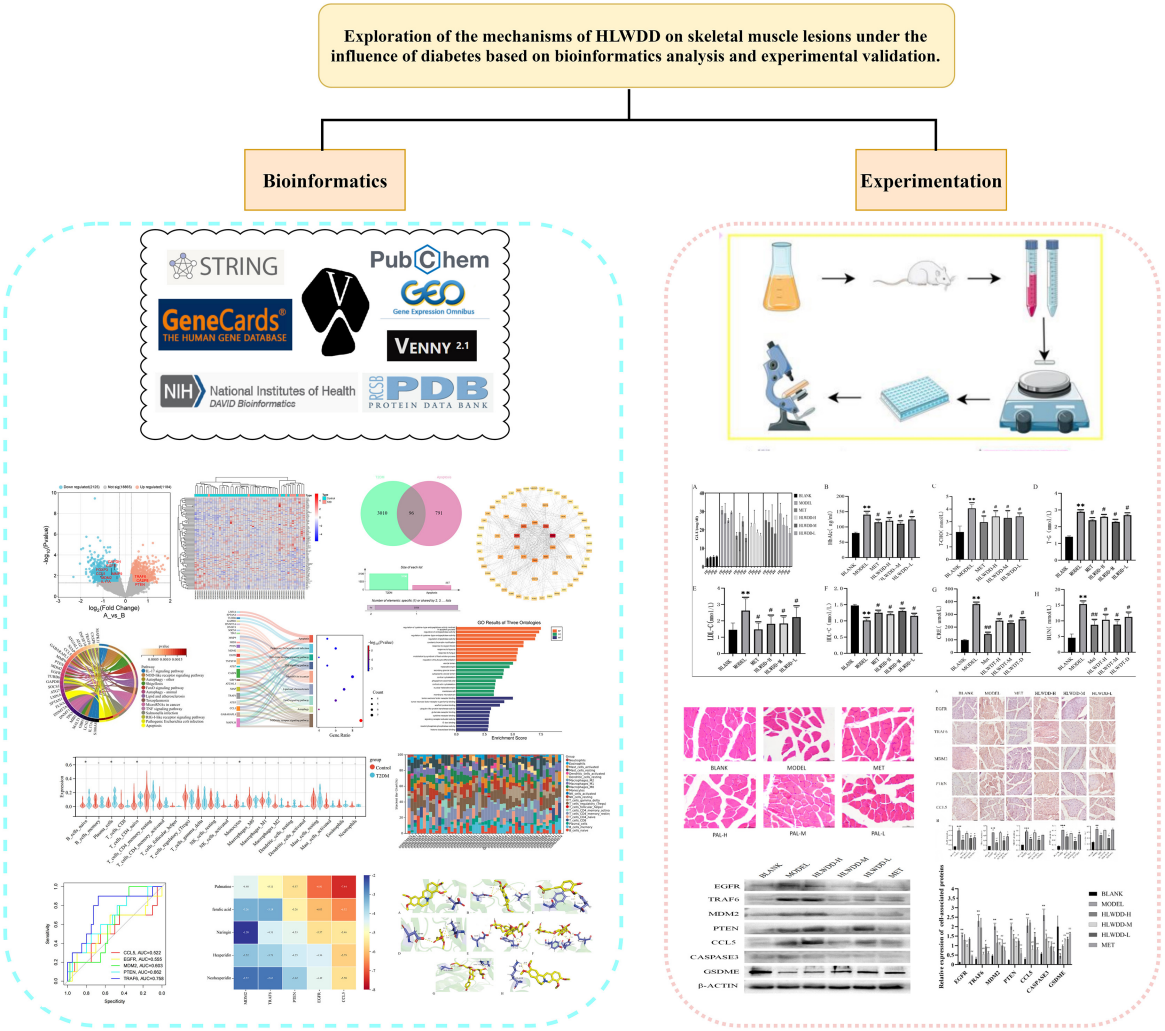
Work flow chart of this study.

Bioinformatics, an interdisciplinary field integrating biology and computational science, has become essential in modern drug discovery and disease mechanism research (39). It employs large-scale data integration and systems-level modeling to uncover complex drug–body interactions and key biological processes in disease. (40). In this study, we applied bioinformatics to investigate the molecular mechanisms of HLWDD in treating T2DM, particularly its role in alleviating skeletal muscle injury. By identifying core target genes and signaling pathways, we aimed to elucidate how HLWDD acts at the molecular level. Although previous studies have reported effects of HLWDD on glucose lowering, islet function, or gut microbiota in T2DM, the novelty of our work lies in three aspects: (1) starting from human skeletal muscle transcriptome data to specifically screen apoptosis-related differentially expressed genes and clarify muscle-level pathological targets; (2) integrating core genes from omics with quantitatively/qualitatively analyzed HLWDD components via molecular docking, establishing a “component–target” evidence chain; and (3) performing *in vivo* validation in HFD+STZ rat models through biochemical, histological, IHC, and Western blot analyses, forming a continuous evidence chain from *in vitro* omics discovery to functional verification. This integrated strategy enhances the credibility and translational potential of HLWDD's mechanism in treating T2DM-related skeletal muscle injury. The findings are expected to provide novel therapeutic insights and identify key molecular targets for future clinical translation (Figure 1).

## 2 Materials and methods

### 2.1 Bioinformatics

#### 2.1.1 Screening differential genes in T2DM

Gene Expression Omnibus (GEO) (http://www.ncbi.nlm.nih.gov/geo/) was searched using the keyword “Type 2 Diabetes” to find microarray datasets from the GEO database, and one diabetes-related dataset (GSE25462) was downloaded. This dataset includes 10 subjects with type 2 diabetes and 40 normal individuals (25 with a family history of type 2 diabetes from one or both parents and 15 without such a history), and the tissue samples in the dataset must come from human skeletal muscle. This dataset was annotated using the Perl software to match probes with platform annotation information and converted probe names to gene names. The expression levels of the probe sets were converted to gene expression levels using the Bioconductor annotation function in R language, averaging the expression values of multiple probes for a specific gene. A gene matrix file was then obtained with the sample names as row names and gene names as column names. Differential analysis was conducted using the limma package in R software (version 4.2.0), The criteria for selecting differentially expressed genes (DEGs) were set as *P* < 0.05 and a dynamic log2FC threshold of |logFC| > [mean(|logFC|) + 2sd(|logFC|)] and volcano plots and heatmaps were generated.

#### 2.1.2 Screening apoptosis-related genes

Using “Apoptosis” as a search term, we collected related target genes from the GeneCards database. Venny 2.1.0 (https://bioinfogp.cnb.csic.es/tools/venny/index.html) was used to identify intersecting target genes between T2DM and Apoptosis, and a Venn diagram was created.

#### 2.1.3 Constructing and visualizing a PPI network

The intersecting target genes of T2DM and Apoptosis were uploaded to the STRING database (https://string-db.org/), with the species option set to “Homosapien” and the minimum interaction threshold set to “Highest confidence > 0.9”. The other parameters were set to default, and a TSV format file was downloaded and imported into Cytoscape 3.9.1. A network of intersecting genes was constructed, core targets were screened based on degree values, and a visualized PPI network diagram was created.

#### 2.1.4 GO and KEGG analysis

The intersecting target genes were subjected to Gene Ontology (GO) and KEGG enrichment analyses using the DAVID database (https://david.ncifcrf.gov/tools.jsp), with *P* < 0.05 as the threshold. The analyses included cellular component (CC), biological process (BP), molecular function (MF), and enriched pathways. Visualization was performed using R.

#### 2.1.5 ROC analysis and visualization

We used the “pROC” package in R to analyze the Receiver Operating Characteristic (ROC) curves for the core genes EGFR, PTEN, MDM2, TRAF6, and CCL5 to evaluate their predictive ability. The analysis was performed in two stages: an initial discovery phase using the GSE25462 dataset, followed by independent validation using the GSE271223 dataset, which includes 24 human subjects (10 healthy controls and 14 with diabetic obesity). For each dataset, the expression data and corresponding sample labels (T2DM vs. healthy) for each gene were extracted. The “roc” function was then used to generate ROC curves for each gene, and their predictive ability was quantified by calculating the area under the curve (AUC). The AUC, as an overall evaluation metric, represents the intrinsic validity of each gene; the closer it is to 1, the better the diagnostic performance. Finally, the ROC curves of all genes were plotted together for each dataset to visually compare their predictive performance. To further assess the clinical relevance, each characteristic gene was evaluated individually in the ROC analysis, with a higher AUC indicating better accuracy in distinguishing disease from normal states.

#### 2.1.6 Immune cell infiltration analysis and immune cell correlation analysis

The obtained DEGs were analyzed for infiltration using the CIBERSORT algorithm with threshold *P* < 0.05 for 22 immune cells, including regulatory T cells, M0 macrophages, activated natural killer cells, etc., using the “e1071, preprocessCore” packages in R. The “corrplot” package was used to generate a heat map of immune cell infiltration correlations, and the “vioplot” package was used to plot the distribution charts of the different immune cell infiltration levels.

#### 2.1.7 Correlation analysis and visualization of characteristic genes and immune cells

Correlation analysis between characteristic genes and immune cells was conducted using the “limma, reshape2, ggpubr, and ggExtra” packages in R. A threshold of *P* < 0.05 indicates a significant correlation between the immune cell and the gene. Correlation analysis was performed using R 4.3.1.

#### 2.1.8 Molecular docking

AutoDock Vina (http://vina.scripps.edu/) was used to dock the chemical components of disease targets. Target proteins were obtained from the PDB database (https://www.rcsb.org/), and their crystal structures were preprocessed. The ligand structures were downloaded from the PubChem database (https://pubchem.ncbi.nlm.nih.gov/). Finally, these target structures were docked with the active component structures using PyRx (https://pyrx.sourceforge.io/), docking was performed with its internal Vina tool, and visualization was performed using PyMOL (https://pymol.org/2/).

### 2.2 Experimental verification

#### 2.2.1 Drugs and reagents

Glycated Hemoglobin (GHb), High-Density Lipoprotein (HDL-C), Low-Density Lipoprotein (LDL-C), Triglycerides (TG), Total Cholesterol (TC), Blood Glucose (GLU), Creatinine (CRE), and Blood Urea Nitrogen (BUN) were obtained from Nanjing Jiancheng Bioengineering Institute. Rabbit Anti-EGFR antibody, Rabbit Anti-PTEN antibody, Rabbit Anti-MDM2 antibody, Rabbit Anti-TRAF6 antibody, and Rabbit Anti-CCL5 antibody kits were acquired from Beijing Bioss Biotechnology Co., Ltd.

#### 2.2.2 Equipment and instruments

The DH-250 electric thermostatic incubator was sourced from Beijing Kewei Yongxing Instrument Co., Ltd. The SpectraMax-190 full-wavelength microplate reader was purchased from Molecular Devices, USA. The ECLIPSE Ni-U Upright Microscope was supplied by Nikon, Japan. The Amersham Imager 680 system for nucleic acid and protein imaging analyses was obtained from GE, USA.

#### 2.2.3 Preparation and quality control of HLWDD

Qualitative analysis of the components of the aqueous extract of HLWDD was performed using liquid chromatography-tandem mass spectrometry. The liquid chromatography equipment consisted of a Shimadzu LC-30AD pump and Shimadzu SPD-20A detector. And the accurate mass spectrometric experiments were operated in the positive and negative ion mode of a TripleTOF 4,600 system with a DuoSpray ion source (AB Sciex, California, USA). 1,086 mg of HuanglianWendan decoction extract was dissolved in 10 ml of solvent (water: ethanol = 1:1), filtered through a 0.22 μm membrane filter, centrifuged at 16,000 × g for 5 min, and the supernatant was collected for analysis. Analysis was carried out on a C18 column (150 mm × 4.6 mm, 3 μm), and the column temperature was maintained at 40 °C. The mobile phase was composed of A (0.1% formic acid in water, v/v) and B (acetonitrile), using a gradient elution of 5% B at 0–2 min, 5–50% B at 2–30 min, 50%−90% B at 30–33 min, 90% B at 33–37 min, and 90%−5% B at 37–37.1 min. The sample injection volume was 5 μl. The flow rate was 0.4 ml/min. The operating parameters were as follows: curtain gas, 30 psi; ion source gas 1 and ion source gas 2, 55 psi; ion spray voltage floating, 5,500 V; temperature, 550 °C; collision energy, 40 V; collision energy spread, 20 V. Data were managed using PeakView software (AB Sciex, California, USA). Quantitative analysis of the above components in the Huanglian-Wendan decoction water extract was performed by high-performance liquid chromatography (HPLC) with a diode array detector (Agilent Technologies 1,200 Series, USA) using an external method. Analysis was carried out on a C18 column (250 mm × 4.6 mm, 5 μm), and the column temperature was maintained at 30 °C. The mobile phase was composed of A (0.1% formic acid in water, v/v) and B (acetonitrile), using a gradient elution of 5% B at 0–2 min, 5%−30% B at 2–10 min, 30%−90% B at 10–20 min, 90% B at 20–35 min, and 90%−5% B at 35–35.1 min. The flow rate was set to 0.8 ml/min. The injection volume for the sample was 2 μl and that for the reference standards was 3 μl. The monitoring wavelengths were set at 210 and 280 nm. Ferulic acid (1.27 mg), naringin (1.97 mg), hesperidin (1.57 mg), neohesperidin (2.03 mg), berberine (2.70 mg), palmatine (2.23 mg), limonin (1.56 mg), and glycyrrhizic acid (2.83 mg) were weighed, respectively. The top five compounds were neohesperidin (2.03 mg), hesperidin (1.57 mg), naringin (1.97 mg), palmatine (2.23 mg), and ferulic acid (1.27 mg) (19). All herbs used complied with the relevant provisions of the 2020 edition of the “Chinese Pharmacopoeia.” The herbs were ground into powder and soaked in 10 times the volume of distilled water for 60 min (1:10 M/W), followed by two extractions at 100 °C for 30 min each. After the first extraction, the liquid medicine was filtered, and 8 times the volume of distilled water was added for further extraction. The two extracts were then combined, vacuum concentrated to 0.78 g·mL^−1^, freeze-dried into a lyophilized powder, and stored at −80 °C for later use.

#### 2.2.4 Grouping and administration

Sixty male Sprague-Dawley (SD) rats, 2 months old (200 ± 20.15 g) were provided by Shanghai BK Lab Animal Ltd. (ID: 20180006053413). The rats were housed in an SPF-grade laboratory for a week of adaptive feeding at a controlled temperature of 25–27 °C and a relative humidity of 50%−70%. Ten rats were randomly selected as the blank group, and the remaining 50 rats were used as the model group to establish the T2DM model. The model group was fed a diet with 45% fat caloric intake for 4 weeks, followed by a day of fasting, and then the rats were administered an intraperitoneal injection of streptozotocin (STZ) (30 mg·kg^−1^). Random blood glucose (RBG) measurements ≥16.7 mmol/L indicated successful T2DM modeling. After modeling, the rats were fed a regular pellet feed for 3 days. The FBG of the blank group was < 6.2 mmol/L, and their 2-h postprandial blood glucose was < 7.9 mmol/L, which met the normal glucose standards. Thirty rats that met the model evaluation criteria were randomly divided into the model group, Metformin Hydrochloride (MET) group (0.04 g·kg^−1^·d^−1^), HLWDD-H group (15.6 mg·kg^−1^·d^−1^), HLWDD-M group (7.8 mg·kg^−1^·d^−1^), and HLWDD-L group (3.9 mg·kg^−1^·d^−1^), with six rats in each group. The dose of HLWDD was administered according to the “Table of Equivalent Dose Ratio Converted by Body Surface Area between Humans and Animals” and the previous study of the reference research group (70). Additionally, six normal rats were selected for the blank group. The administration volume was 10 ml·kg^−1^, and the rats in the normagroup and the model group were given the same volume of distilled water by gavage for 4 weeks.

#### 2.2.5 Biochemical testing

Serum samples from each group of rats were used to measure HbA1c, HDL-C, LDL-C, TC, TG, GLU, BUN, and CRE levels. Blood collected from the abdominal aorta was centrifuged at 15,000 rpm for 10 min and the supernatant was extracted. The absorbance was measured using a microplate reader according to the manufacturer's instructions, and the serum concentrations of HbA1c, HDL-C, LDL-C, TC, TG, GLU, BUN, and CRE were calculated.

#### 2.2.6 HE staining

Dissected rat skeletal muscle tissues were fixed in formalin solution. After fixation, the tissues were dehydrated in a graded alcohol series, cleared in xylene, and embedded in paraffin. Paraffin-embedded tissues were then sectioned, deparaffinized, hydrated, and stained with hematoxylin and eosin. The sections underwent successive alcohol dehydration and were fully clarified in xylene before being mounted with a resin. Observations and photographs were taken under a microscope.

#### 2.2.7 Immunohistochemical staining

Immunohistochemical staining was used to detect the levels of EGFR, PTEN, MDM2, TRAF6, and CCL5 in the skeletal muscle tissues of the rats in each group. After deparaffinization and gradual alcohol treatment, paraffin sections were rinsed with running water for 5 min and immersed in a 3% hydrogen peroxide solution to block endogenous peroxidase, followed by antigen retrieval with citrate buffer. They were then washed three times with PBS for 5 min each. Primary antibodies against EGFR, PTEN, MDM2, TRAF6, and CCL5 were added and the cells were incubated overnight at 4 °C. The following day, after washing with PBS, a secondary antibody was added and incubated for 2 h, followed by another PBS wash step. DAB was added, followed by washing with PBS. The sections underwent graded alcohol dehydration and were mounted with a resin. Observations and photographs were taken under a microscope. The positively stained area in five randomly selected fields from each sample was measured using ImageJ software. The average positive area percentage was calculated for each rat (*n* = 6 per group) and used for statistical analysis.

#### 2.2.8 Western blot analysis

Skeletal muscle tissues were added to RIPA buffer in an Eppendorf tube and centrifuged at 12,000 × g for 5 min at 4 °C to collect the supernatant. The protein concentration was determined using a BCA kit. Eppendorf tubes containing the extracted proteins were thawed on ice. The sample buffer was added to achieve the same loading volume, and the samples were boiled for 5–10 min to denature the proteins before loading. Twenty micrograms of sample was subjected to electrophoresis and membrane transfer, followed by blocking with 5% skim milk. Primary antibodies against EGFR, PTEN, MDM2, TRAF6, and CCL5 were added and the cells were incubated overnight at 4 °C. The PVDF membrane was then incubated with HRP-labeled secondary antibodies at room temperature for 1 h. Finally, ECL reagent was used for development, and images were captured using a gel imaging system. The images were analyzed using β-actin as an internal control, and the ratio of the grayscale values of the target protein to the internal control protein was calculated using the ImageJ software.

## 3 Results

### 3.1 Bioinformatics

#### 3.1.1 Screening differential genes in T2DM

**Figure 2:**
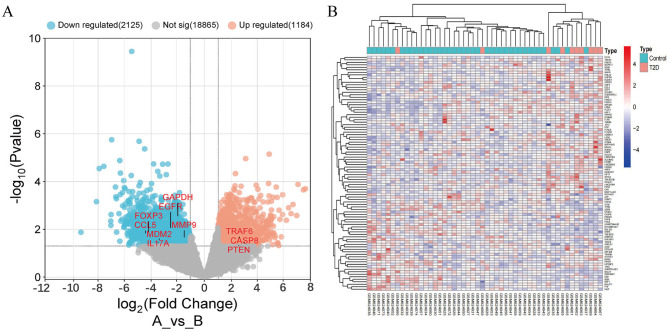
The transcriptomic profiles of patients with type 2 diabetes and healthy controls were analyzed. **(A)** Volcano plot showing significantly differentially expressed genes (DEGs) between type 2 diabetes and healthy samples in the GSE25462 dataset. **(B)** Heatmap displaying the significantly upregulated and downregulated DEGs in the GSE25462 dataset.

The GSE25462 dataset was analyzed using the Limma package in R, The dynamic log2FC threshold for GSE25462 was set at 1.0, and volcano and heat maps (Figure 2) were generated based on differentially expressed genes between the type 2 diabetes and healthy control groups. A total of 3,309 differentially expressed genes were identified in the GSE25462 dataset, including 1,184 upregulated and 2,125 downregulated genes (Figures 2A, B).

#### 3.1.2 Screening apoptosis-related genes and potential disease targets

**Figure 3:**
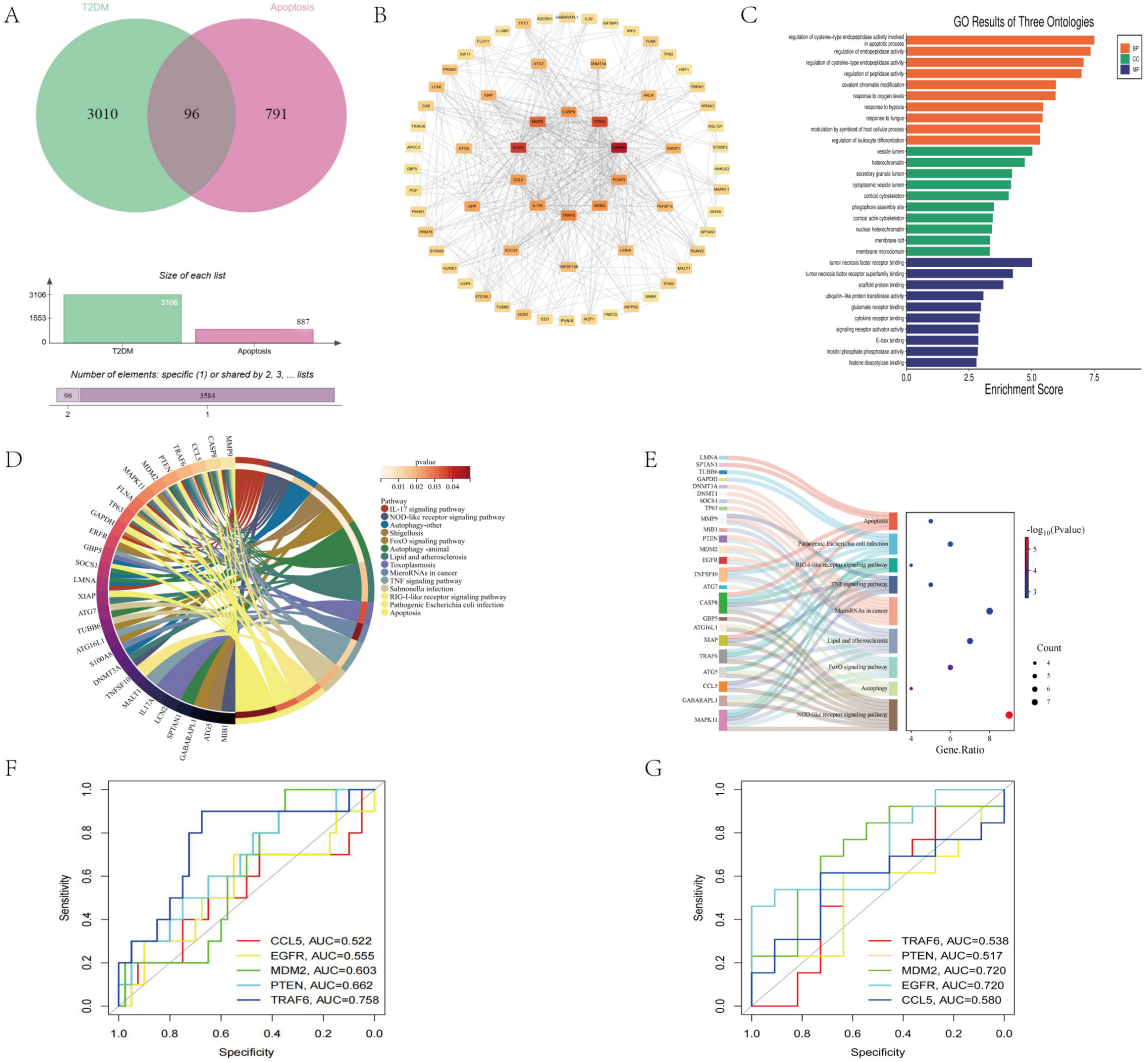
**(A)** Venn diagram showing Apoptosis and T2DM. **(B)** PPI analysis of the intersecting target genes. The top 10 hub genes were GAPDH, EGFR, PTEN, MMP9, TRAF6, CASP8, MDM2, FOXP3, IL17A, and CCL5. **(C)** GO analysis. **(D)** KEGG analysis. **(E)** Sankey diagram of “core target pathways. **(F)** ROC curves of the core genes. TRAF6 (AUC = 0.758), PTEN (AUC = 0.662), MDM2 (AUC = 0.603), EGFR (AUC = 0.555), and CCL5 (AUC = 0.522). **(G)** ROC validation curve of the core genes. TRAF6 (AUC = 0.538), PTEN (AUC = 0.517), MDM2 (AUC = 0.720), EGFR (AUC = 0.720), and CCL5 (AUC = 0.580).

Using “Apoptosis” and “T2DM” as keywords, we retrieved 887 apoptosis-related and 3,106 T2DM-related target genes from GeneCards. Venny 2.1.0 identified 96 intersecting genes (Figure 3A), which were analyzed via the STRING database to construct a protein-protein interaction network (80 nodes, 247 edges). This network was visualized using Cytoscape 3.9.1, and the CytoHubba plugin was used to identify the top 10 core targets by degree centrality: GAPDH, EGFR, PTEN, MMP9, TRAF6, CASP8, MDM2, FOXP3, IL17A, and CCL5 (Figure 3B). Functional enrichment analysis using DAVID revealed 1,019 significant GO terms (*P* < 0.05), including 884 biological processes (e.g., regulation of cysteine-type endopeptidase activity in apoptosis), 66 cellular components (e.g., vesicle lumen and heterochromatin), and 69 molecular functions (Figure 3C). KEGG analysis highlighted 172 pathways, with IL-17, NOD-like receptor, autophagy, FoxO, and apoptosis pathways containing the highest concentration of core genes (Figure 3D). A Sankey diagram further elucidated the core gene-pathway relationships (Figure 3E). ROC analysis confirmed the diagnostic value of the five core genes for T2DM: TRAF6 (AUC = 0.758), PTEN (AUC = 0.662), MDM2 (AUC = 0.603), EGFR (AUC = 0.555), and CCL5 (AUC = 0.522) (Figure 3F). To validate these findings, an independent validation analysis was performed using the GSE271223 dataset. Results showed that the five core genes consistently exhibited significant diagnostic efficacy, with the area under the receiver operating characteristic curve (AUC) values as follows: TRAF6 (AUC = 0.538), PTEN (AUC = 0.517), MDM2 (AUC = 0.720), EGFR (AUC = 0.720), and CCL5 (AUC = 0.580) (Figure 3G). These observations further confirm the robustness of our primary discovery.

#### 3.1.3 Immune infiltration analysis and visualization

**Figure 4:**
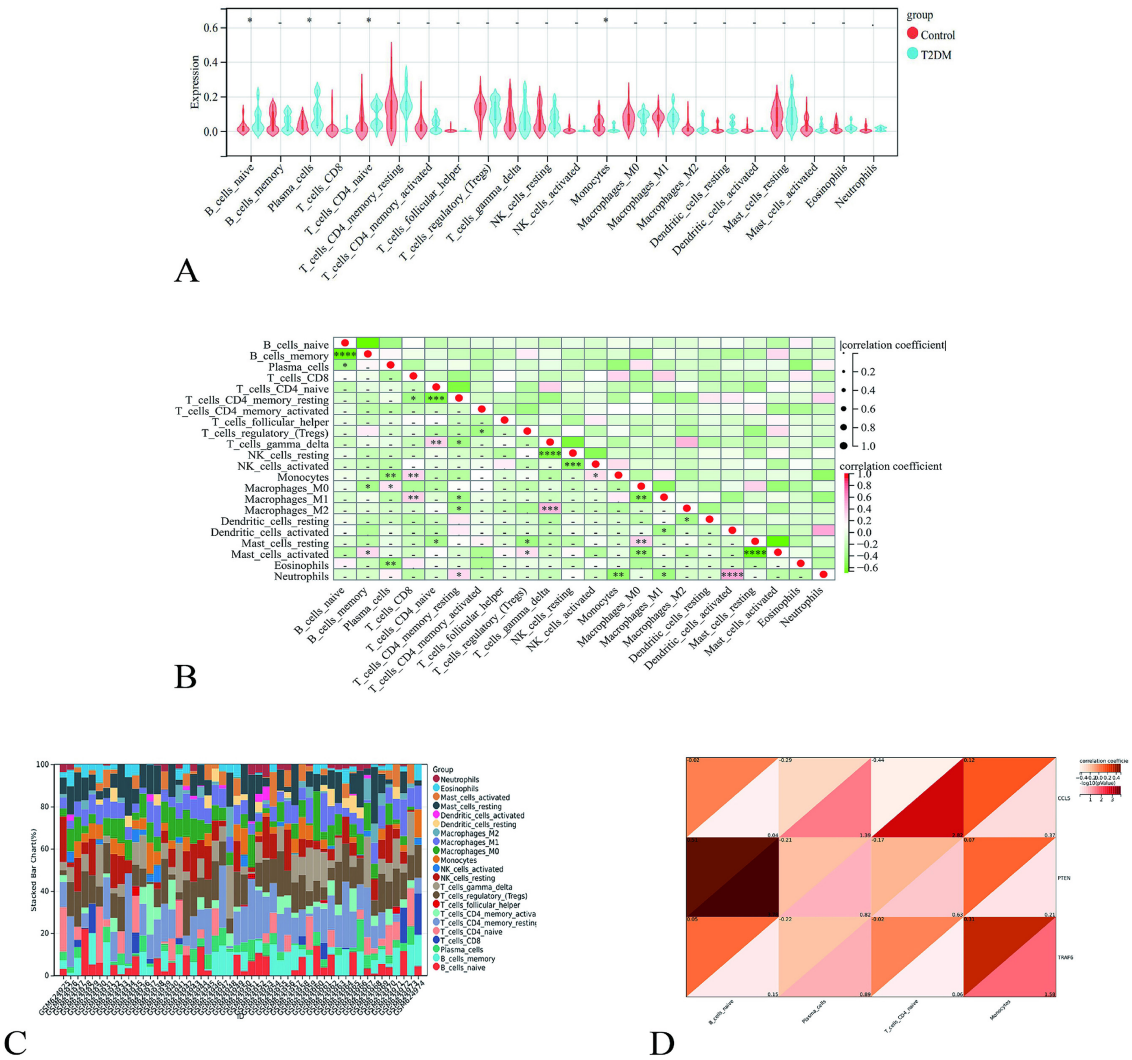
Immune infiltration analysis and visualization. **(A)** Violin plot. **(B)** Immune cell correlation heatmap. **(C)** Stacked bar chart of the immune cell distribution. **(D)** Heat map of correlations between core genes and immune cells.

The violin plot shows that in skeletal muscle, the proportions of immune cells, such as naive B cells (*P* < 0.05) and plasma cells (*P* < 0.05), were higher in the model group than in the blank group, while naive CD4 T cells (*P* < 0.05) and monocytes (*P* < 0.05) were lower in the model group than in the blank group (Figure 4A). The immune cell correlation heatmap reveals that naive B cells were negatively correlated with B cells memory (*R* = −0.64, *P* < 0.01) and plasma cells (*R* = −0.32, *P* < 0.05). Plasma cells were negatively correlated with Monocytes (*R* = −0.36, *P* < 0.01) and Eosinophils (*R* = −0.37, *P* < 0.01) but positively correlated with Macrophages_M0 (*R* = 0.3, *P* < 0.05). Naive CD4 T cells were negatively correlated with T cells CD4 memory_resting (*R* = −0.52, *P* < 0.01) and Mast cells_resting (*R* = −0.29, *P* < 0.05) but positively correlated with T cells_gamma_delta (*R* = 0.38, *P* < 0.01). Monocytes were positively correlated with T cells CD8 (*R* = 0.37, *P* < 0.01) and NK cells activated (*R* = 0.32, *P* < 0.05) but negatively correlated with plasma cells (*R* = −0.36, *P* < 0.01) (Figure 4B). The distribution of immune cells in each sample is shown as a stacked bar chart (Figure 4C). The heatmap of core genes and immune cells showed that CCL5 was negatively correlated with plasma cells (*R* = −0.29, –log10 *P* > 1.3) and T cells CD4 memory_resting (*R* = −0.44, –log10 *P* > 1.3). PTEN was positively correlated with naive B cells (*R* = 0.51, –log10 *P* > 1.3), whereas TRAF6 was positively correlated with Monocytes (*R* = 0.31, –log10 *P* > 1.3) (Figure 4D).

#### 3.1.4 Molecular docking

**Figure 5:**
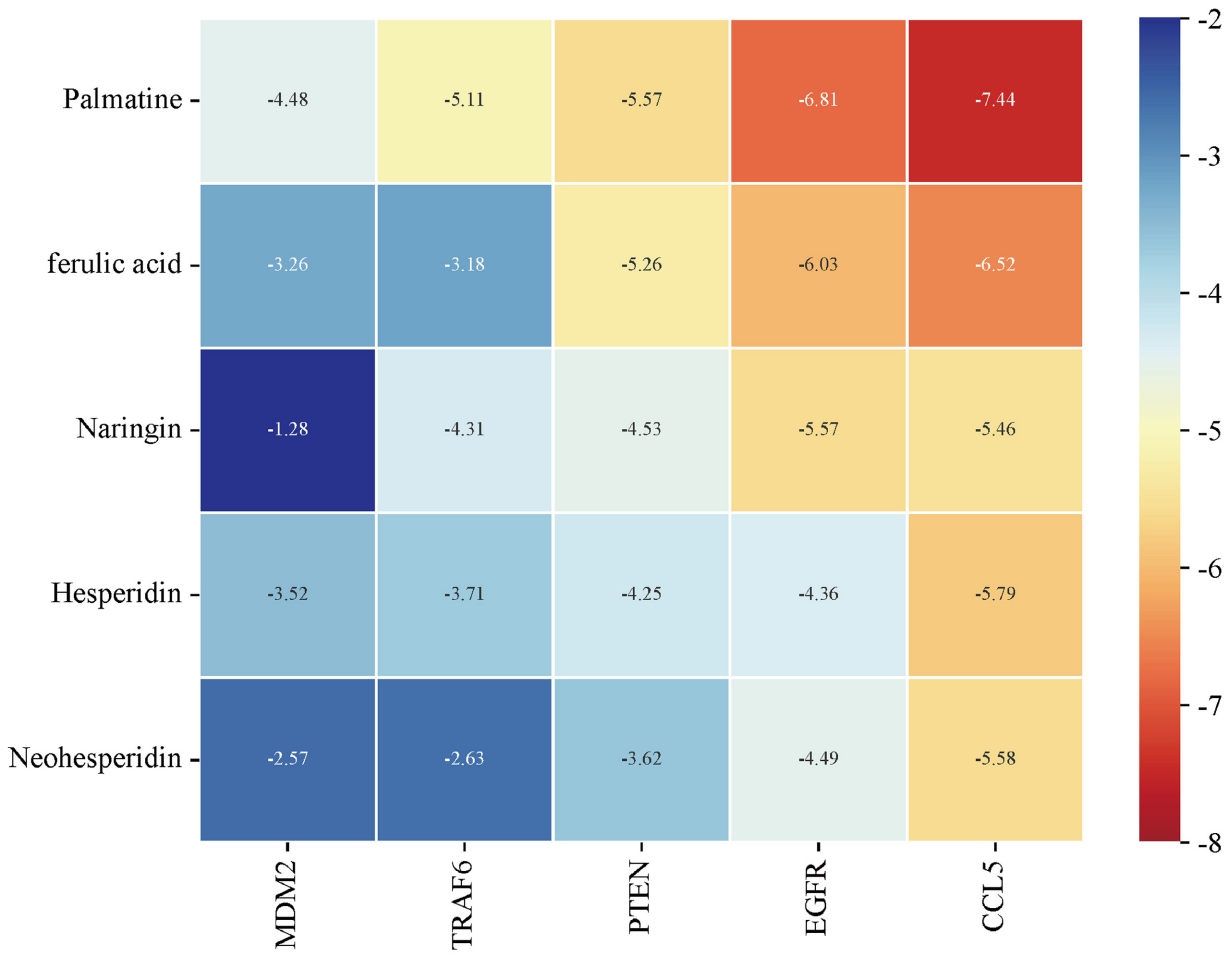
Heatmap of molecular docking binding energies. The top five compounds were docked with the target proteins, with lower binding energies represented by blue modules and higher binding energies represented by red modules.

**Figure 6:**
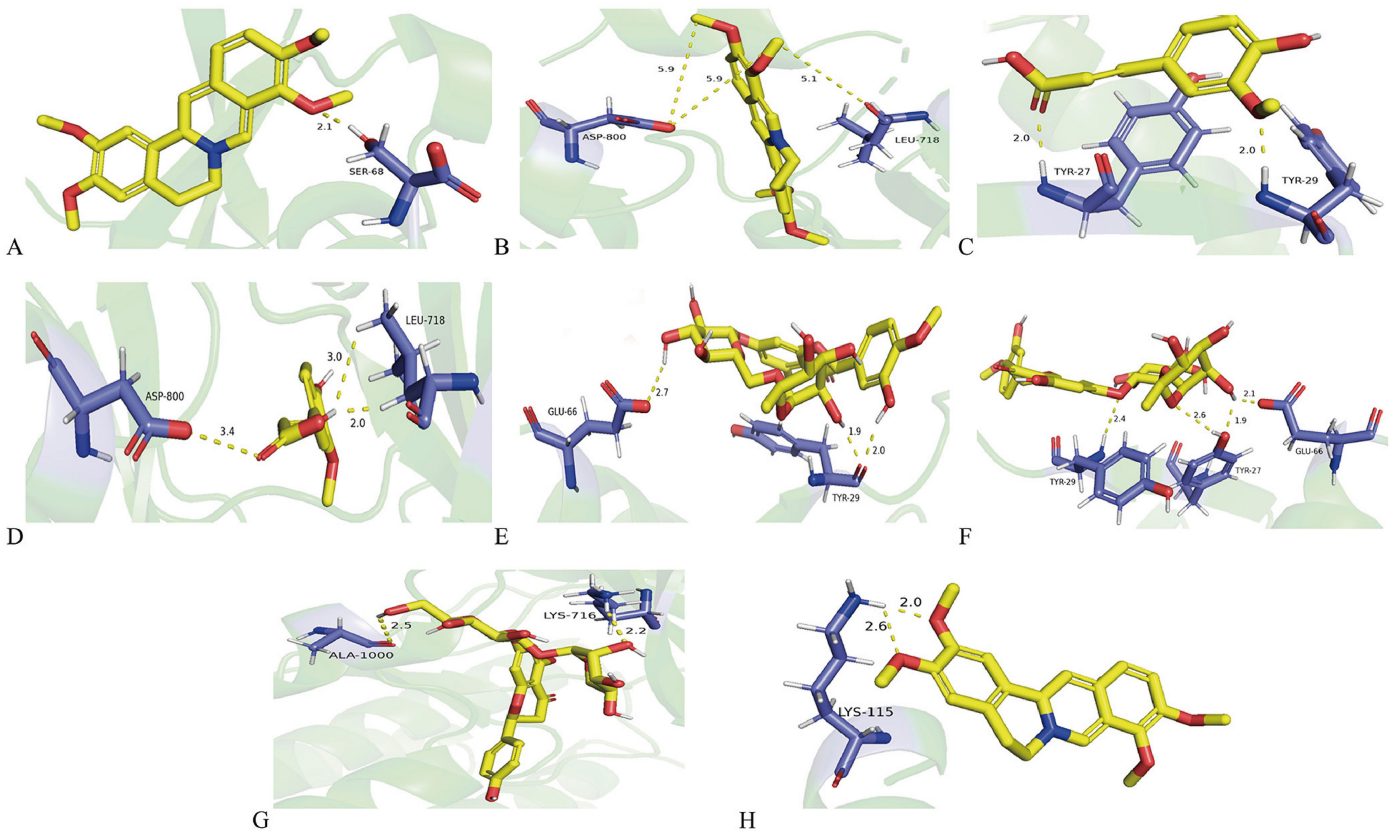
Molecular docking of core targets and chemical components. **(A)** Palmatine-CCL5. **(B)** Palmatine-EGFR. **(C)** Ferulic acid-CCL5. **(D)** Ferulic acid-EGFR. **(E)** Hesperidin-CCL5. **(F)** Neohesperidin-CCL5. **(G)** Naringin-EGFR. **(H)** Palmatine-PTEN.

To further verify the therapeutic effects of HLWDD on T2DM, the top five core chemical components, neohesperidin (2.03 mg), hesperidin (1.57 mg), naringin (1.97 mg), palmatine (2.23 mg), and ferulic acid (1.27 mg), were docked with the five core target proteins (Figure 5). The binding stability between the ligand and receptor is negatively correlated with the binding energy; generally, a lower binding energy indicates a better binding interaction. The top eight binding interactions based on binding energy were visualized using molecular docking diagrams (Figure 6).

### 3.2 Experimental verification results

#### 3.2.1 Effect of HLWDD on glucose and lipid metabolism and renal function in T2DM model rats

**Figure 7:**
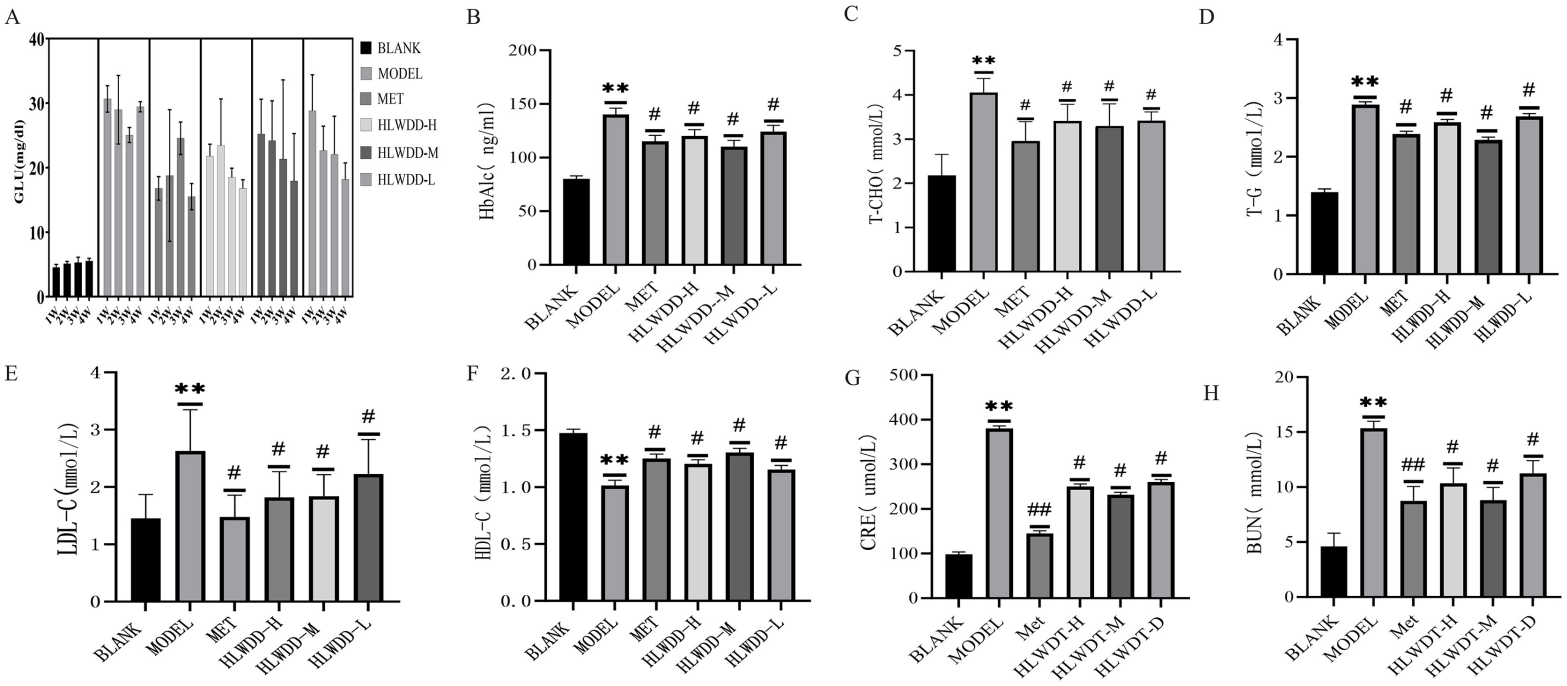
Levels of glucose, lipids, and renal function in each group of rats. **(A)** Blood glucose. **(B)** HbA1c. **(C)** T-C. **(D)** T-G. **(E)** LDL-C. **(F)** HDL-C. **(G)** CRE. **(H)** BUN. *n* = 6. **P < 0.01 compared with the blank group; ^#^*P* < 0.05, ^##^*P* < 0.01 compared with the model group. Detailed statistical data (Mean ± SD and *P*-values) are available in Supplementary Table S1.

The effectiveness of T2DM treatment is primarily indicated by changes in the blood glucose and lipid levels. To assess the efficacy of HLWDD in treating T2DM, we observed changes in blood glucose and lipid levels in each group of rats through biochemical tests. The results showed that the HbA1c, LDL-C, TC, TG, and GLU levels of the model group were significantly higher than those of the blank group, whereas HDL-C was significantly lower (Figures 7A–F). However, after intervention with Met and HLWDD, the blood glucose and lipid levels in the rats were significantly reduced compared with those in the model group. Additionally, to evaluate the impact of HLWDD on renal function in rats, we measured BUN and CRE (Figures 7G, H). Satisfyingly, HLWDD did not have a significant effect on renal function in any of the groups, as BUN and CRE levels remained within the normal range.

#### 3.2.2 Effect of HLWDD on the pathological morphology of skeletal muscle in T2DM model rats

**Figure 8:**
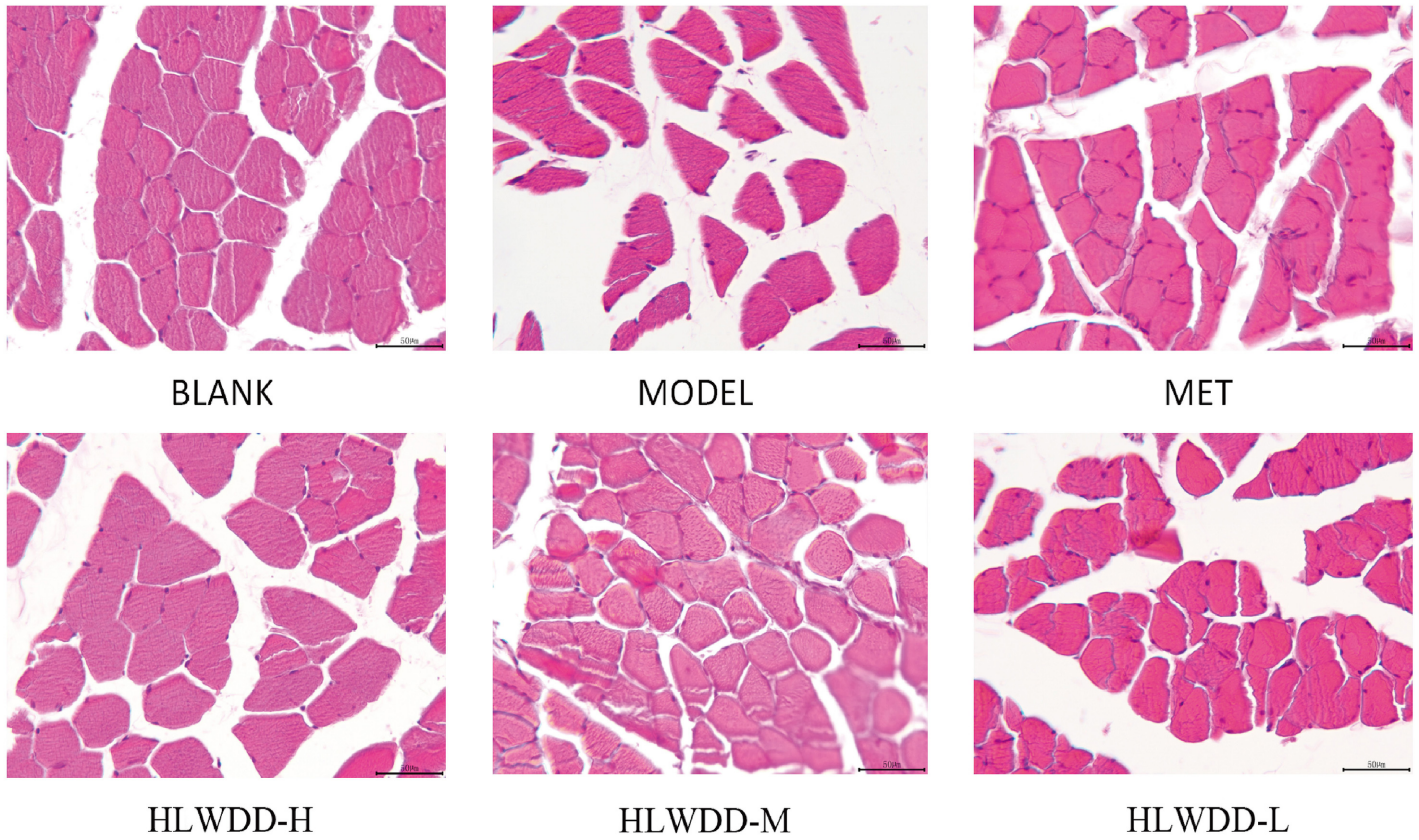
HE staining. skeletal muscle HE staining, × 200.

To observe the effect of HLWDD on the pathological morphology of skeletal muscle in T2DM models, HE staining was performed on the skeletal muscle of each group of rats (Figure 8). The results showed that the skeletal muscle cells of the blank group rats were regularly polygonal and densely arranged, with nuclei located at the cell edges and muscle bundles clearly and neatly demarcated. In contrast, the skeletal muscle cells of the model group rats were loosely arranged, severely deformed with atrophic changes, and some cells were hypertrophic with interstitial edema. However, the structure of skeletal muscle tissue in rats improved after HLWDD intervention, although a small number of necrotic cells remained. Additionally, no significant difference was observed between the skeletal muscle tissue of rats after Met intervention and that of rats in the HLWDD group.

#### 3.2.3 Effect of HLWDD on the levels of EGFR, TRAF6, MDM2, PTEN, and CCL5 in skeletal muscle of T2DM model rats

**Figure 9:**
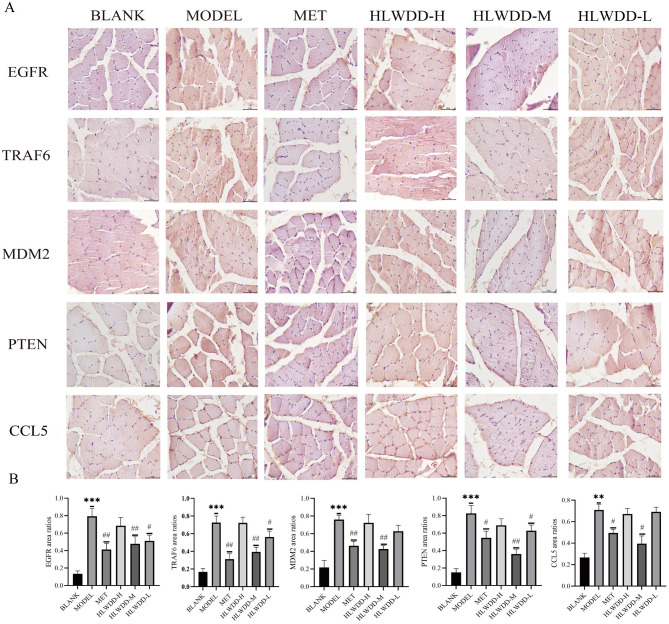
IHC staining. **(A)** IHC staining of EGFR, TRAF6, MDM2, PTEN, and CCL5 in skeletal muscle ( × 200). **(B)** Quantitative analysis of the positive staining areas for EGFR, TRAF6, MDM2, PTEN, and CCL5 in the skeletal muscle. *n* = 6. **P < 0.01, ***P < 0.001 compared with the blank group; ^#^*P* < 0.05, ^##^*P* < 0.01 compared with the model group.

IHC staining was performed to evaluate the expression levels of five key proteins—EGFR, TRAF6, MDM2, PTEN, and CCL5—in the rat skeletal muscle tissues (Figure 9A). The staining results revealed distinct expression patterns of these proteins across experimental groups (Figure 9B). The blank group exhibited the lowest expression levels of all five proteins. In contrast, marked upregulation was observed in the model group, suggesting that the modeling procedure likely activated signaling pathways associated with inflammation or apoptosis regulation. Notably, the administration of HLWDD at high-, medium-, and low-dose levels suppressed the model-induced overexpression of these proteins to varying degrees. Among the HLWDD-treated groups, the HLWDD-M showed a trend toward the most pronounced regulatory effect, with protein expression levels significantly lower than those in the model group and lower than those in both the HLWDD-H and HLWDD-L groups. This result implies that the efficacy of HLWDD may be dose dependent within a certain range, with the medium dose demonstrating a particularly strong therapeutic trend in our experimental setting. Furthermore, the protein expression levels in the MET group were comparable to those in the HLWDD-M group. Both groups showed significantly lower expression than the levels in the model group approached those observed in the blank group, indicating that the two interventions elicited similar effects in modulating the expression of these key proteins.

#### 3.2.4 Effect on the protein levels of EGFR, TRAF6, MDM2, PTEN, and CCL5 in T2DM model rats

**Figure 10:**
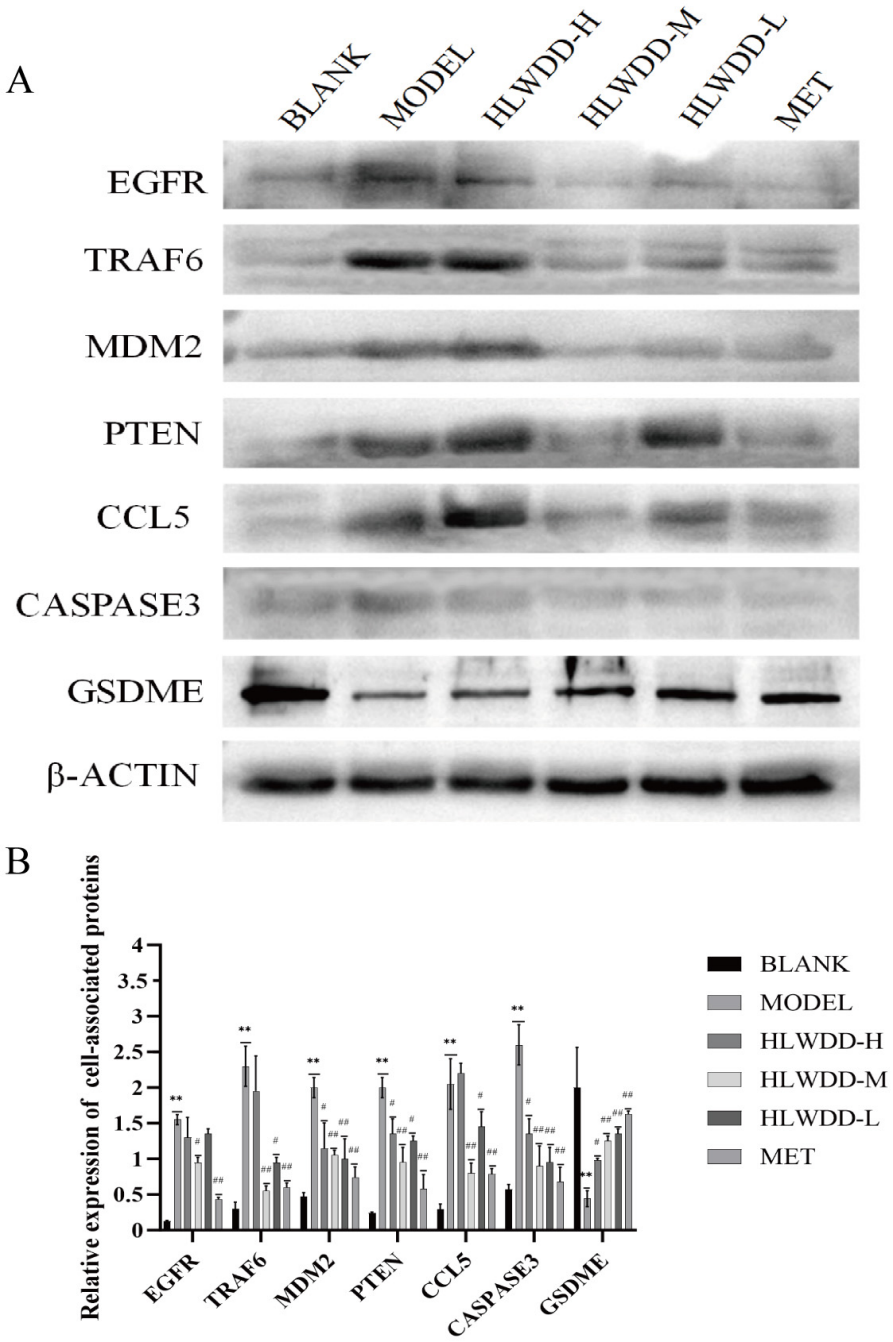
Western Blot. **(A)** Western blot analysis of EGFR, TRAF6, MDM2, PTEN, CCL5, Caspase-3, and GSDME proteins in the skeletal muscle. **(B)** Quantitative analysis of the protein expression levels of EGFR, TRAF6, MDM2, PTEN, CCL5, Caspase-3, and GSDME. *n* = 6. **P < 0.01 compared with the blank group; ^#^*P* < 0.05, ^##^*P* < 0.01 compared with the model group.

In previous experiments, we assessed the expression of EGFR, TRAF6, MDM2, PTEN, and CCL5 by immunohistochemistry. To further characterize protein expression, we conducted western blot analysis to determine the protein levels of EGFR, TRAF6, MDM2, PTEN, and CCL5 in the skeletal muscle tissues of each group of rats, as well as the expression of the apoptosis-related markers Caspase-3 and GSDME (Figure 10A). The results showed that the protein expression levels of EGFR, TRAF6, MDM2, PTEN, CCL5, and the apoptosis marker Caspase-3 were significantly higher in the model group rats compared to the blank group, and GSDME was significantly higher in the blank group. After intervention with Met, HLWDD-H, HLWDD-M, and HLWDD-L groups, the expression levels of EGFR, TRAF6, MDM2, PTEN, and CCL5 proteins were significantly reduced, and the apoptosis marker Caspase-3 was also decreased compared to the model group, while GSDME was increased compared to the model group (Figure 10B). The slightly higher CCL5 expression in the HLWDD-H group than in the model group was not statistically significant (*P* > 0.05), possibly due to individual differences.

## 4 Discussion

Diabetes is a metabolic disorder characterized primarily by insufficient or impaired insulin secretion, leading to elevated blood glucose levels over an extended period (41). The core pathological mechanism of T2DM is insulin resistance, where body cells become less sensitive to insulin, making it difficult to regulate blood glucose levels and resulting in hyperglycemia. Poor lifestyle habits, such as lack of exercise and high-calorie diets, are significant factors that contribute to the development of T2DM (42). As the most common type of diabetes, T2DM is a complex multifactorial disease often culminating in severe complications such as diabetic myopathy, retinopathy, and nephropathy (43). The pharmacological armamentarium against T2DM, including mainstays like metformin and SGLT2 inhibitors, has largely been developed around a “single-target, high-potency” paradigm, effectively addressing specific physiological pathways such as hepatic gluconeogenesis or renal glucose reabsorption (44). While indispensable, this reductionist approach can be limited by off-target effects, compensatory mechanisms, and an inability to fully address the disease's intertwined pathophysiological cores—chronic inflammation, metabolic dysregulation, and cellular demise—often leaving complications unmitigated and leading to side effects like gastrointestinal issues or fluid retention (45). In this context, the polypharmacology of Traditional Chinese Medicine (TCM) formulations like HLWDD presents a compelling alternative strategy. (38, 46–49). Rather than opposing, HLWDD's action can be viewed as complementary and systems-oriented, potentially filling the therapeutic gaps left by single-target drugs. Our study, alongside others, suggests that HLWDD does not merely lower glucose but orchestrates a broad-spectrum restoration of metabolic homeostasis. For example, while metformin activates AMPK in the liver, HLWDD constituents like naringin and quercetin appear to activate AMPK in peripheral tissues like skeletal muscle, thereby promoting glucose uptake and mitigating insulin resistance through a parallel yet distinct pathway (50, 51). More significantly, HLWDD concurrently engages targets beyond the scope of most conventional glucose-lowering drugs. Its documented suppression of the TLR4/NF-κB and NLRP3/caspase-1 axes directly counteracts the chronic low-grade inflammation that underpins insulin resistance (18, 19). Furthermore, its remodeling of the gut microbiota toward a beneficial landscape (e.g., promoting Prevotella and Bifidobacterium) introduces a holistic, systems-level intervention that modulates host metabolism from outside the classical insulin signaling cascade (52). The therapeutic profile of HLWDD emerges from the synergistic interplay of its phytochemical consortium. This includes berberine for enhancing glycolysis (53); β-sitosterol and kaempferol for potentiating insulin signaling and GLUT4 translocation (54–56); and ferulic acid for combating oxidative stress (30). HLWDD confers protection against diabetic muscle injury by downregulating apoptosis and immune-related targets, an effect unmet by first-line drugs. This multi-target action allows it not only to lower blood glucose but also to “cool inflammation” and “reinforce cellular resilience.” Consequently, HLWDD complements conventional drugs by simultaneously managing metabolic symptoms and mitigating tissue damage, offering a more holistic therapeutic strategy.

While the above evidence confirms that HLWDD exerts therapeutic effects on T2DM through multi-component, multi-pathway regulation—including anti-inflammation, gut-microbiota modulation, and improvement of glucose-lipid metabolism (57),—the precise molecular targets and downstream signaling networks, particularly those involved in alleviating T2DM-related skeletal-muscle lesions, remain unclear. To address this gap, we applied integrated bioinformatics analyses to identify potential core regulatory molecules by intersecting T2DM-related differentially expressed genes (DEGs) with apoptosis-associated proteins—a process closely linked to T2DM-induced tissue injury—and validated them using receiver-operating-characteristic (ROC) curves. This approach identified five key genes—EGFR, PTEN, MDM2, TRAF6, and CCL5—which may mediate HLWDD's therapeutic effects, laying the groundwork for elucidating its molecular mechanisms.

GO and KEGG analyses showed that these core targets are mainly associated with apoptotic and inflammatory signaling, particularly the NOD-like receptor, FoxO, TNF, and autophagy pathways. Caspase-3, a cysteine protease widely recognized as a hallmark of apoptosis, is a principal executioner of programmed cell death (58, 59). Upon activation by intrinsic or extrinsic apoptotic cues, Caspase-3 cleaves downstream substrates, resulting in cellular disassembly. When Caspase-3 cleaves gasdermin E (GSDME)—the product of a tumor-suppressor gene—its N-terminal fragment forms membrane pores, causing cellular swelling and rupture; conversely, low GSDME expression leads to classical apoptosis (60). These mechanisms determine whether cells undergo apoptosis or pyroptosis, thereby influencing the extent of tissue injury in diabetes. The epidermal growth factor receptor (EGFR, also known as ErbB1/HER1) is a member of the receptor-tyrosine-kinase ErbB family, contains a transmembrane and an intracellular kinase domain. Upon activation, EGFR autophosphorylates tyrosine residues that act as docking sites for signaling proteins within the MAPK, JAK/STAT, Src, and PI3K pathways—cascades controlling proliferation, differentiation, and apoptosis (61). EGFR is thus considered a potential target in diabetes-related complications (62). PTEN, a dual-specificity phosphatase, hydrolyzes PIP3 to PIP2, thereby negatively regulating the PI3K-AKT pathway (63). Because this pathway acts downstream of the insulin receptor and IRS proteins, PTEN plays a pivotal role in glucose metabolism. The PI3K-AKT axis facilitates insulin-mediated glucose uptake and GLUT4 translocation by inhibiting the GAP protein AS160 (TBC1D4) (64). Thus, excessive PTEN expression impairs insulin signaling and contributes to insulin resistance (65). MDM2, an E3 ubiquitin ligase localized in both nucleus and cytoplasm, regulates p53 stability and is involved in apoptosis, the cell cycle, and the DNA-damage response (66). Over-stabilized MDM2 induces cytoplasmic accumulation of NDUFS1, disrupting mitochondrial super-complex assembly, enhancing ROS production, and initiating BIM-mediated BAK/BAX-dependent apoptosis—events that lead to insulin resistance (67). Tumor necrosis factor receptor-associated molecule 6 (TRAF6), a key adaptor in inflammatory signaling, mediates activation of the TRAF6/TAK1/NF-κB cascade in response to hyperglycemia-induced immune stimulation, thereby promoting TNF-α, IL-1β, and IL-6 expression (71), This pathway contributes to diabetic renal fibrosis and functional impairment through epithelial-mesenchymal transition (EMT). Chemokine ligand 5 (CCL5), also known as regulated upon Activation, Normal T Cell Expressed and Secreted (RANTES), a C-C chemokine binding its receptor CCR5 on T cells and macrophages (68), is up-regulated in diabetic serum, islets, and kidneys, where it promotes inflammation and β-cell injury (69). Our *in vivo* experiments further demonstrated that HLWDD reduced serum LDL-C, TC, TG, GLU, BUN, and CRE levels in T2DM model rats while down-regulating EGFR, PTEN, MDM2, TRAF6, CCL5, and Caspase-3 expression. These results align with previous findings and suggest that HLWDD alleviates T2DM-associated skeletal-muscle damage primarily by suppressing apoptosis-related and inflammatory signaling pathways.

## 5 Conclusion

This study clarified the previously limited understanding of how Huanglian Wendan Decoction (HLWDD) alleviates skeletal-muscle injury associated with type 2 diabetes mellitus (T2DM). By integrating bioinformatics analyses with *in vivo* experimental validation, we identified five core apoptosis- and immunity-related targets—EGFR, PTEN, MDM2, TRAF6, and CCL5—that are potentially modulated by the bioactive compounds of HLWDD. HLWDD markedly improved glucose- and lipid-metabolism indices as well as skeletal-muscle histopathology in T2DM rats, most likely through coordinated regulation of apoptotic and immune-infiltration pathways. These findings highlight the multi-component, multi-target, and multi-pathway characteristics of HLWDD, providing mechanistic evidence for its therapeutic potential in metabolic disorders such as T2DM. However, we used GeneCards for apoptosis- and T2DM-related gene screening; while GeneCards is comprehensive, a single aggregated database may risk “circular reasoning”: data heterogeneity and cross-referencing may over-enrich widely reported genes, missing new/under-annotated targets. Though PPI, ROC, and *in vivo* experiments enhanced reliability, future studies need independent databases for cross-validation to reduce bias. Additionally, the use of a single T2DM rat model and lack of long-term toxicity/pharmacokinetic assessments limit findings' generalizability. More mechanistic and clinical studies are needed. Future research should focus on clinical translation, dosage optimization, safety assessment, and multi-database/animal model strategies to advance HLWDD's evidence-based use in diabetes management.

## Data Availability

The original contributions presented in the study are included in the article/Supplementary material, further inquiries can be directed to the corresponding author.
