# Supplementary material for: Exploration of the mechanisms of HLWDD on skeletal muscle lesions under the influence of diabetes based on bioinformatics analysis and experimental validation
Source: Front Nutr. 2025 Dec 11;12:1586761. doi: 10.3389/fnut.2025.1586761 (PMC12739385; doi:10.3389/fnut.2025.1586761)
Supplement: Supplementary file 1 [file Table_1.docx]

| **LDL-C** | **Mean±SD** |  | **P-value** |
| --- | --- | --- | --- |
| BLANK | 1.45±0.10 | BLANK vs MODEL | 0.007 |
| MODEL | 2.75±0.12 | MODEL vs MET | 0.021 |
| MET | 1.57±0.11 | MODEL vs HLWDD-H | 0.036 |
| HLWDD-H | 1.88±0.12 | MODEL vs HLWDD-M | 0.018 |
| HLWDD-M | 1.85±0.10 | MODEL vs HLWDD-L | 0.048 |
| HLWDD-L | 2.18±0.11 |  |  |

| **T-G** | **Mean±SD** |  | **P-value** |
| --- | --- | --- | --- |
| BLANK | 1.39±0.05 | BLANK vs MODEL | 0.005 |
| MODEL | 2.89±0.05 | MODEL vs MET | 0.029 |
| MET | 2.39±0.05 | MODEL vs HLWDD-H | 0.031 |
| HLWDD-H | 2.59±0.05 | MODEL vs HLWDD-M | 0.021 |
| HLWDD-M | 2.29±0.05 | MODEL vs HLWDD-L | 0.038 |
| HLWDD-L | 2.69±0.05 |  |  |

| **HDL-C** | **Mean±SD** |  | **P-value** |
| --- | --- | --- | --- |
| BLANK | 1.47±0.03 | BLANK vs MODEL | 0.003 |
| MODEL | 1.01±0.05 | MODEL vs MET | 0.023 |
| MET | 1.25±0.04 | MODEL vs HLWDD-H | 0.040 |
| HLWDD-H | 1.20±0.04 | MODEL vs HLWDD-M | 0.021 |
| HLWDD-M | 1.30±0.03 | MODEL vs HLWDD-L | 0.043 |
| HLWDD-L | 1.15±0.04 |  |  |

| **HbA1c** | **Mean±SD** |  | **P-value** |
| --- | --- | --- | --- |
| BLANK | 80±3 | BLANK vs MODEL | 0.004 |
| MODEL | 140±8 | MODEL vs MET | 0.033 |
| MET | 115±7 | MODEL vs HLWDD-H | 0.038 |
| HLWDD-H | 120±8 | MODEL vs HLWDD-M | 0.023 |
| HLWDD-M | 108±6 | MODEL vs HLWDD-L | 0.046 |
| HLWDD-L | 124±7 |  |  |

| **T-C** | **Mean±SD** |  | **P-value** |
| --- | --- | --- | --- |
| BLANK | 2.20±0.10 | BLANK vs MODEL | 0.006 |
| MODEL | 4.09±0.16 | MODEL vs MET | 0.025 |
| MET | 3.28±0.13 | MODEL vs HLWDD-H | 0.036 |
| HLWDD-H | 3.48±0.13 | MODEL vs HLWDD-M | 0.017 |
| HLWDD-M | 3.18±0.12 | MODEL vs HLWDD-L | 0.048 |
| HLWDD-L | 3.68±0.13 |  |  |

| **CRE** | **Mean±SD** |  | **P-value** |
| --- | --- | --- | --- |
| BLANK | 97±5 | BLANK vs MODEL | 0.007 |
| MODEL | 380±6 | MODEL vs Met | 0.009 |
| Met | 145±6 | MODEL vs HLWDD-H | 0.032 |
| HLWDD-H | 260±6 | MODEL vs HLWDD-M | 0.015 |
| HLWDD-M | 240±6 | MODEL vs HLWDD-L | 0.044 |
| HLWDD-L | 270±6 |  |  |

| **BUN** | **Mean±SD** |  | **P-value** |
| --- | --- | --- | --- |
| BLANK | 4.5±0.3 | BLANK vs MODEL | 0.005 |
| MODEL | 15.0±0.6 | MODEL vs Met | 0.006 |
| Met | 9.7±0.6 | MODEL vs HLWDD-H | 0.035 |
| HLWDD-H | 10.7±0.6 | MODEL vs HLWDD-M | 0.016 |
| HLWDD-M | 8.7±0.6 | MODEL vs HLWDD-L | 0.047 |
| HLWDD-L | 12.7±0.6 |  |  |
